# Monty Roberts’ Public Demonstrations: Preliminary Report on the Heart Rate and Heart Rate Variability of Horses Undergoing Training during Live Audience Events

**DOI:** 10.3390/ani6090055

**Published:** 2016-09-09

**Authors:** Loni Loftus, Kelly Marks, Rosie Jones-McVey, Jose L. Gonzales, Veronica L. Fowler

**Affiliations:** 1 Askham Bryan College, York YO23 3FR, UK; loni.loftus@askham-bryan.ac.uk; 2 Intelligent Horsemanship Ltd., Lethornes, Lambourn, Hungerford, Berkshire RG17 8QP, UK; kelly@ihhq.net; 3 The Old Schools, University of Cambridge, Trinity Lane, Cambridge CB2 1TN, UK; rosiejoneshorses@yahoo.co.uk; 4 Department of Epidemiology, Crisis Organization and Diagnostics, Central Veterinary Institute Part of Wageningen UR, Houtribweg 39, Lelystad 8221, The Netherlands; jose.gonzales@wur.nl; 5 The Pirbright Institute, Ash Road, Pirbright, Surrey GU24 0NF, UK

**Keywords:** horse training, Monty Roberts, live demonstration, heart rate, heart rate variability

## Abstract

**Simple Summary:**

Monty Roberts is a famous horse trainer, commonly referred to as a ‘horse whisperer’, who shares his training methods all over the world, including through large public audience events. These events have the potential to compromise the horse’s welfare since the horses have usually been transported to the event on the day and stabled in an unfamiliar environment before being used in demonstrations watched by hundreds of people. This paper describes the opportunistic collection and analysis of heart rate (HR; beat-to-beat intervals) and heart rate variability (HRV) of horses being trained during Monty Roberts’ public demonstrations within the United Kingdom. HR and HRV measured during the demonstrations were lower (indicative of an increase in heart rate as measured in beats-per-minute) than recordings within the stable and consistent with low-moderate exercise intensities used during training. The HR and HRV during a specific training method known as “Join-up^®^” were comparable to other methods of training used by Monty Roberts during public demonstrations. In conclusion, training of horses during public demonstrations is a low-moderate physiological, rather than psychological stressor for horses, with the stress response comparable or less than those previously reported in the literature for horses being trained outside of public audience events. Furthermore, we found no evidence that Join-up^®^ alters HR and HRV in a way to suggest that this training method negatively affects the psychological welfare of horses.

**Abstract:**

Effective training of horses relies on the trainer’s awareness of learning theory and equine ethology, and should be undertaken with skill and time. Some trainers, such as Monty Roberts, share their methods through the medium of public demonstrations. This paper describes the opportunistic analysis of beat-to-beat (RR) intervals and heart rate variability (HRV) of ten horses being used in Monty Roberts’ public demonstrations within the United Kingdom. RR and HRV was measured in the stable before training and during training. The HRV variables standard deviation of the RR interval (SDRR), root mean square of successive RR differences (RMSSD), geometric means standard deviation 1 (SD1) and 2 (SD2), along with the low and high frequency ratio (LF/HF ratio) were calculated. The minimum, average and maximum RR intervals were significantly lower in training (indicative of an increase in heart rate as measured in beats-per-minute) than in the stable (*p* = 0.0006; *p* = 0.01; *p* = 0.03). SDRR, RMSSD, SD1, SD2 and the LF/HF ratio were all significantly lower in training than in the stable (*p* = 0.001; *p* = 0.049; *p* = 0.049; *p* = 0.001; *p* = 0.01). When comparing the HR and HRV of horses during Join-up^®^ to overall training, there were no significant differences in any variable with the exception of maximum RR which was significantly lower (*p* = 0.007) during Join-up^®^, indicative of short increases in physical exertion (canter) associated with this training exercise. In conclusion, training of horses during public demonstrations is a low-moderate physiological, rather than psychological stressor for horses. The physiological stress responses observed within this study were comparable or less to those previously reported in the literature for horses being trained outside of public audience events. Furthermore, there is no evidence that the use of Join-up^®^ alters HR and HRV in a way to suggest that this training method negatively affects the psychological welfare of horses.

## 1. Introduction

The effective training of horses is a practice which should be based on learning theory and equine ethology and undertaken with skill and time [1]. Horse training techniques, which are considered more sympathetic than traditional approaches, have been well reported to result in less fear responses and in some cases produce horses with greater technical performance [1,2,3,4]. The Monty Roberts method is one of those techniques reported to be more efficacious when compared to traditional approaches used for the initial training of horses over a three week period [2].

Monty Roberts and his accredited trainers routinely tour the world sharing these methods with large audiences during public demonstrations. During these events there is potential for the horse’s welfare to be compromised by fear responses since the horses have usually been transported to the event on the day and stabled in an unfamiliar environment before being used in short, method intensive training sessions.

It is possible to measure the stress response of horses both physiologically and behaviourally, however, during initial training (first rider; first saddle) and remedial training (desensitization of hyper-reactive behaviour), such as those used in Monty Roberts’ public demonstrations, horses are less able to display behaviour in the same way as unhandled horses. In circumstances such as these, it is commonly accepted to use heart rate (HR) and heart rate variability (HRV) as physiological stress parameters, either on their own or in combination with salivary cortisol. HRV reflects the balance between the sympathetic and parasympathetic tone of the autonomic nervous system and has been suggested as a valuable tool for assessing autonomic modulation of cardiac activity at low-moderate exercise [5] assuming careful interpretation of the data [6,7]. Adaptability of the horse to changing environments is governed by a close interplay between the parasympathetic nervous system (PNS) and sympathetic nervous system (SNS). Parasympathetic influences reduce HR, whilst sympathetic influences increase HR, however, the total effect of both these branches of the nervous system on HR, can be a result of either synchronous or independent influence [6].

HR and/or HRV (using time-domain analysis and frequency-domain analysis) have been used to define the welfare and quantify the degree of psychological stress for horses undergoing various tasks, including initial training [2,8,9,10], desensitization to alarming stimuli [11], public competition [12,13], ridden obstacle tests [14], forced-backward movement [15], novel object/startle tests [16,17], transportation [18,19], and sudden individual stabling [20], all of which are possible events which horses used in Monty Roberts’ public demonstrations may experience. In addition, during these demonstrations Monty Roberts uses a method called Join-up^®^ [21] which has been considered by some as fear evoking via hypothesized overt activation of the flight response [22]. The term “Join-up^®^” is a training method defined by Monty Roberts, which is claimed to result in the development of a human-horse relationship/bond where the horse considers interaction with the human trainer as safe. Scientifically, Join-up^®^ can be described as the application and withdrawal of pressure, typical of negative reinforcement. In the case of Join-up^®^, the application of pressure involves the horse trainer moving the horse away from them, freely, around the outside of a round pen using visual (strong eye contact and square “assertive” body language and perhaps including various other methods such as flicking of ropes, hand movements) and audio cues (verbal noises such as clucking/squeaking to encourage movement). During this phase, the trainer will keep this pressure on while looking for certain behaviours such as head-lowering, licking and chewing, inside ear directed towards the trainer and the horse reducing the distance between itself and the trainer (smaller circles). Once these behaviours are observed, all pressure is removed with the expected outcome that the horse then willingly approaches the trainer and will freely follow him or her around the round pen (the latter described by Monty Roberts as follow up^®^ [21]).

Currently, there are no studies which have investigated the HR and HRV of horses undergoing training during public demonstrations such as those organized by Monty Roberts. The purpose of this paper was to undertake a preliminary study to determine the HR and HRV of horses used in Monty Roberts’ public demonstrations, specifically looking at the response of the horses within the stable before training, during training and during Join-up^®^ and follow-up^®^.

## 2. Materials and Methods

### 2.1. Ethical Approval

The data collected during this study was opportunistically collected (e.g., monitoring of horses which were going to be used as part of independently pre-arranged public demonstrations) and was approved by independent ethical review committees at the participating institutions (A/B/P/230516). Informed consent was obtained from all owners prior to inclusion in this study following the provision of detailed participant information and withdrawal details. Inclusion criteria specified that all horses had been confirmed fit and well by appointed independent Veterinarians and Veterinary Physiotherapists. Heart rate monitors were only fitted to horses which were familiar with being touched around the girth area. The authors did not augment the procedures of the demonstrations in any way other than to collect participant information and consent, and to fit and remove the heart rate monitors.

### 2.2. Horses and Training Classifications

Ten horses comprising seven geldings and three mares of a range of breeds and ages were used in this study (Table 1). Horses were used to demonstrate application of first saddle and rider (*n* = 3) or methods to overcome remedial problems (*n* = 7). Within the remedial classification, methods were used to resolve hyper-reactivity: (a) loading refusal (*n* = 2); (b) spooky behaviour (*n* = 2); (c) ear shyness (*n* = 1); (d) bike shyness (*n* = 1), and mounting refusal (*n* = 1). Eight horses had arrived on the day of the show, whilst two horses were already present for more than one week. Transportation times between home and the demonstration locations for the eight horses brought on the day of the show ranged between 30–90 min. All horses had arrived at the demonstration locations and had been stabled, on straw bedding, with free movement and *ad libitum* hay for more than one hour before being monitored for this study. The stables were located in barns from which each demonstration horse could see a range of other horses (all unfamiliar). Five of the ten horses had been in an arena before, two horses had been in a round pen before and four horses had experienced events with small audiences. None of the horses had worn a heart rate monitor before.

### 2.3. Location and Video Recording of Demonstrations

The demonstrations were conducted at Reaseheath College, Hartpury College, and Hadlow College within indoor schools of the following sizes; Reaseheath (30 m × 60 m), Hartpury (70 m × 40 m), and Hadlow (60 m × 35 m). At each location the audience size was 930, 907, and 898 respectively. The round pen used for the training of all horses was situated as close as possible to the centre of the arena. For the horses used to demonstrate methods to resolve loading refusal, training began in the round pen but also involved using a larger area of the arena into which an Oakley horsebox was located. Each horse was video recorded from the moment it entered the round pen. Horses were not video recorded within the stables.

### 2.4. Trainers, Riders and Owner Involvement

Horses were trained by Monty Roberts (MR) (*n* = 9), or MR in combination with Kelly Marks (KM) (*n* = 1; bike shy horse). Horses being used to demonstrate first rider were mounted and ridden by the same assistant. A second rider was used to ride two remedial horses (bike shy and mounting refusal). Owners were involved at the end of three training sessions (loading and mounting refusal).

### 2.5. Training Schedule

For each horse, the training steps and total training time varied (Table 2). In the context of this paper, “centaur training” comprised MR riding a companion horse within the round pen whilst leading and touching the ears of the remedial horse. Training involving the SR1 Ardall dummy (Ardall, Ireland), starting with the dummy in the “down” position, progressing to the addition of the weighted legs, before elevating the dummy to the “upright” position. The term “Join-Up^®^” defines the period of time from when the horse was unclipped from the lead rope and underwent “Join-Up^®^” and “follow-up^®^” [21].

### 2.6. Heart Rate and Heart Rate Variability

To measure heart rate (HR) and heart rate variability (HRV), two identical mobile recording systems were used (Polar RS800CX; Polar Electro Oy, Kempele, Finland). The data was analysed using Polar Pro Trainer (version 5.42.002, Kempele, Finland). The custom filter was set to maximum for artefact correction in RR (ms). The program automatically calculated minimum (min), average (ave) and maximum (max) RR intervals (ms), standard deviation of the RR interval (SDRR), root mean square of successive RR differences (RMSSD) and the geometric means standard deviation 1 (SD1) and 2 (SD2). In the frequency domain the program computed LF (0.04–0.15 Hz), HF (0.15–0.40 Hz) and LF/HF ratio, consistent with a range of papers studying frequency domain in horses [14,15,23,24]. The stopwatch on the polar RS800CX was synchronized with the time on the video recorder so that the HR and HRV data could be accurately matched to the training steps. Due to the nature of the demonstration and opportunistic data collection, it was not possible to standardize the length of the recording times for both time in stable and time during training sessions. Recording times captured for the horses within the stable and overall training session are shown in Table 2.

### 2.7. Statistical Analysis

Generalized linear mixed models (GLMM) were used to determine the difference between stable and overall training and between Join-Up^®^ and specific training, where horses were included as random effects, HR and HRV values as response variables and the variables of interest for comparison (e.g., location of the horse, training type, sex) as the explanatory variables. The inclusion of each horse as random effect accounted for the repeated measures made on the same animal, for example first in the stable and then during training (paired observations). Although GLMM are robust to departures from assumptions of normality and constant variance, normality of the response variables (RR, SDRR, RMSSD, SD1, SD2, LF/HF ratio) used for comparison was assessed using the Shapiro-Wilk test. For variables which were not normally distributed, the GLMM outcomes were further confirmed using the Wilcoxon signed-rank test with concordance observed between these tests. The threshold for statistical significance was set to *p* < 0.05. The “specific training” terminology refers to all training excluding “Join-Up^®^”.

## 3. Results

### 3.1. Effect of Overall Training on Heart Rate and Heart Rate Variability during an Audience Demonstration

**Figure 1:**
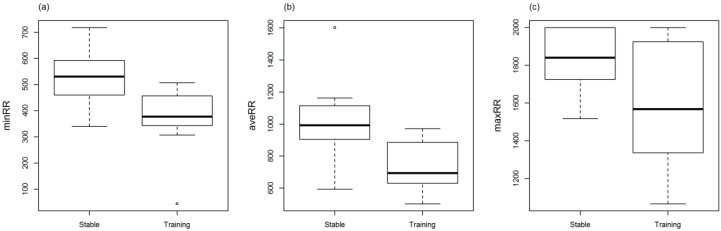
Minimum (**a**), average (**b**), and maximum (**c**) beat-to-beat (RR) intervals (ms) of horses before training (Stable) and during Monty Roberts’ public demonstration (Training). RR intervals were significantly lower in training than in the stable (minimum: *p* = 0.0006, average: *p* = 0.01, maximum *p* = 0.03). Analysed using generalized linear mixed models (GLMM).

**Figure 2:**
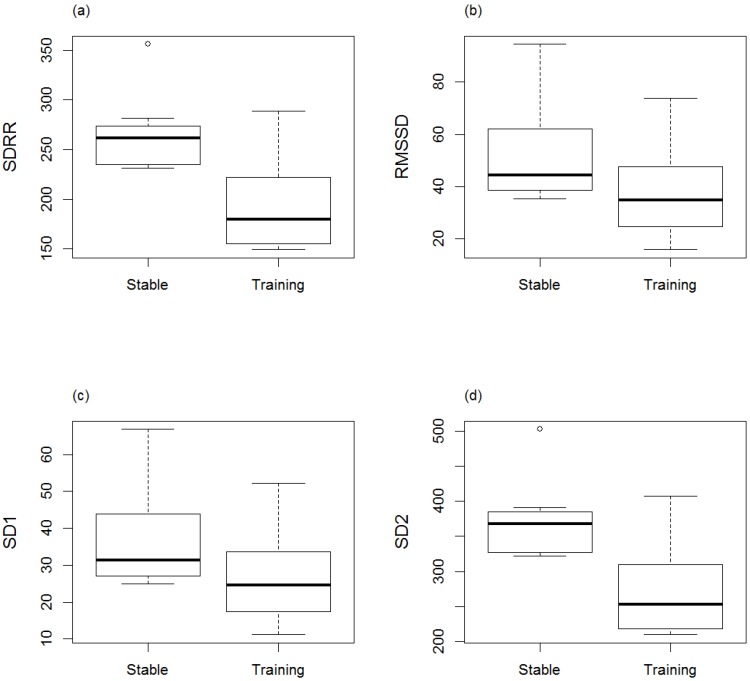
Standard deviation of the RR interval (SDRR) (**a**), root mean square of successive RR differences (RMSSD) (**b**), and the geometric means standard deviation 1 (SD1) (**c**) and 2 (SD2) (**d**) of horses before training (Stable) and during Monty Roberts’ public demonstrations (Training). SDRR, RMSSD and geometric means SD1 and SD2 were significantly lower in training (RR: SDRR: *p* = 0.001; RMSSD: *p* = 0.0489; SD1: *p* = 0.0489; SD2: *p* = 0.001). Analysed using generalized linear mixed models (GLMM) and the Wilcoxon signed-rank test.

**Figure 3:**
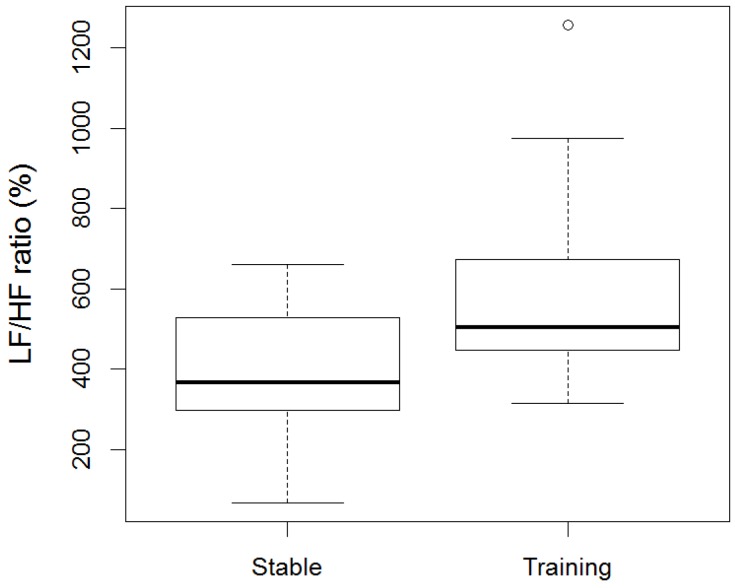
LF/HF ratio of horses before training (Stable) and during Monty Roberts’ public demonstrations (Training). The LF/HF ratio was significantly higher (*p* = 0.01) in training when compared to stable. Analysed using generalized linear mixed models (GLMM) and Wilcoxon signed-rank test.

Minimum, average, and maximum HR (bpm) within the stable were (mean ± SD) 33.4 ± 3.66, 62.8 ± 16.85, and 120.9 ± 29.39, respectively, and within training were (mean ± SD) 39.4 ± 8.09, 85.1 ± 16.64, and 157.8 ± 28.71, respectively. RR intervals, SDRR, RMSSD, and geometric means SD1 and SD2 were significantly lower in training (RR: min, ave, max: *p* = 0.0006, *p* = 0.01, *p* = 0.03; SDRR: *p* = 0.001; RMSSD: *p* = 0.049; SD1: *p* = 0.049; SD2: *p* = 0.001) compared to the stable (Figure 1a–c, Figure 2a–d). The LF/HF ratio was significantly higher (*p* = 0.01) in training compared to the stable (Figure 3). There was no difference in RR, LF/HF ratio, SDRR, SD1, SD2, and RMSSD between starters or remedial horses and no effect of sex, or demonstration location.

### 3.2. Effect of First Join-Up^®^ on Heart Rate and Heart Rate Variability

**Figure 4:**
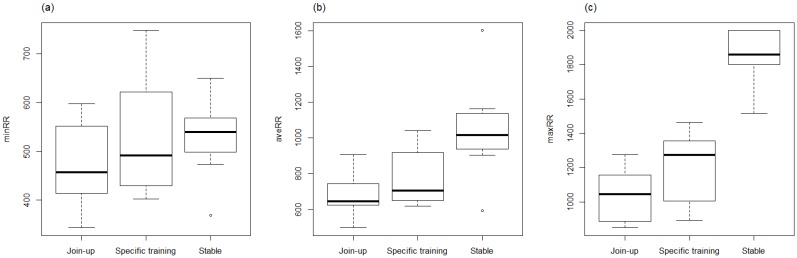
Minimum (**a**), average (**b**), and maximum (**c**) beat-to-beat (RR) intervals (ms) of horses before training (Stable), during their first Join-Up^®^ (Join-up) and during the rest of the training session (Specific training). RR intervals were significantly lower in Join-up^®^ (*p* = 0.007) than in specific training. Analysed using generalized linear mixed models (GLMM).

**Figure 5:**
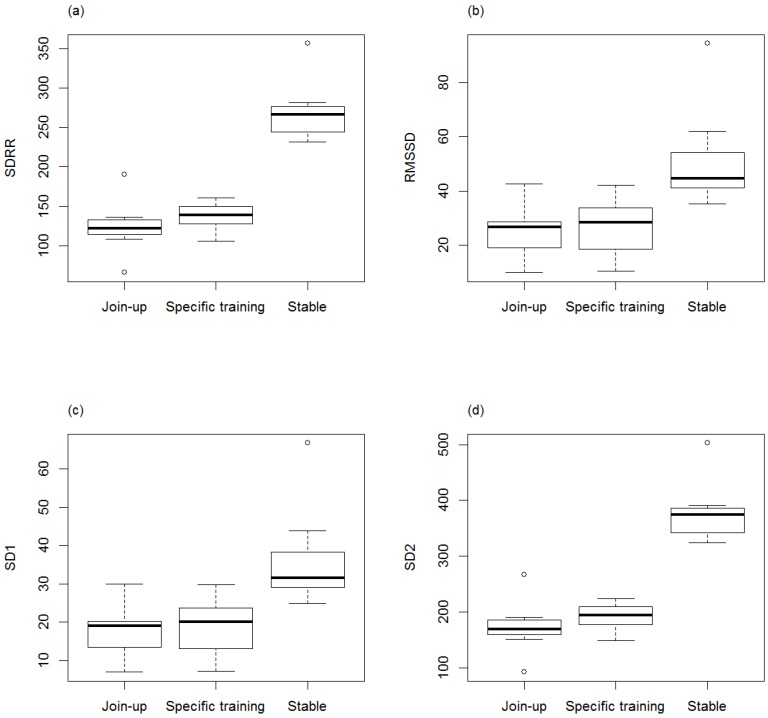
Standard deviation of the RR interval (SDRR) (**a**), root mean square of successive RR differences (RMSSD) (**b**), and the geometric means standard deviation 1 (SD1) (**c**) and 2 (SD2) (**d**) before training (Stable), during their first Join-Up^®^ (Join-up) and during the rest of the training session (Specific training). No statistical difference was observed between first Join-Up^®^ (Join-up) and during the rest of the training session (Specific training). Analysed using generalized linear mixed models (GLMM) and the Wilcoxon signed-rank test.

**Figure 6:**
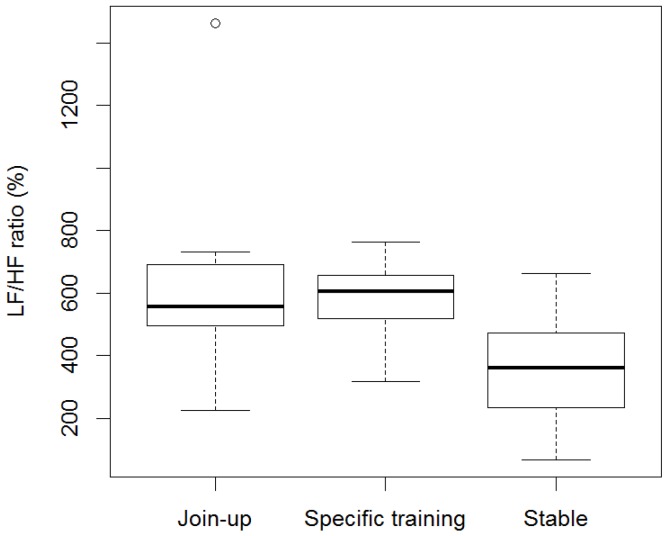
LF/HF ratio of horses before training (Stable), during their first Join-Up^®^ (Join-up) and during the rest of the training session (Specific training). No statistical difference was observed between first Join-Up^®^ (Join-up) and during the rest of the training session (Specific training). Analysed using generalized linear mixed models (GLMM) and the Wilcoxon signed-rank test.

Minimum, average and maximum HR (bpm) during first “Join-Up^®^” were (mean ± SD) 50.86 ± 15.70, 87.14 ± 15.72, and 130.29 ± 26.80, respectively, and within the rest of the training session (specific training-excluding any further Join-up^®^’s) were (mean ± SD) 51.57 ± 10.90, 77.05 ± 14.14, and 123.43 ± 21.38, respectively. Minimum and average RR intervals during first “Join-Up^®^“ were not significantly different to the rest of the training session (Figure 4a–c). Maximum RR intervals were significantly lower (*p* = 0.007) during first “Join-Up^®^” than during the rest of the training session. There was no difference in LF/HF ratio, SDRR, SD1, SD2, and RMSSD between first Join-Up^®^ and the rest of the training session (Figure 5a–d and Figure 6). Where outliers were observed, these tended to correlate with the same horse(s). For example, the low outlier for SDRR and SD2 and the high outlier for LF/HF ratio in Join-Up^®^ was horse E. The high outlier for RMSSD and SD1 in the stable was horse H. The high outlier for SDRR and SD2 in the stable was horse B.

## 4. Discussion

This paper describes the opportunistic analysis of RR intervals and HRV of ten horses being used in Monty Roberts’ public demonstrations within the United Kingdom. RR intervals and HRV (SDRR, RMSSD, SD1, SD2, and LF/HF ratio) were measured in the stable before training, during overall training, and during a training method known as Join-up^®^.

Baseline RR intervals within the stable were lower (i.e., HRs marginally higher) than baseline values from other studies [5,18,19,23,24] but remained higher (i.e., HRs marginally lower) than those measured in horses anticipating competition [25]. The lower baseline values reported in this paper most likely reflect the fact that, in all but two cases, the horses had travelled to the demonstration location no more than 180 min before the start of the training and were, therefore, more unsettled than if the horses had been in a familiar environment before the demonstration.

The minimum, average, and maximum RR intervals were significantly lower during overall training and during Join-up^®^ compared to the stable. Maximum RR intervals were also significantly lower during Join-up^®^ compared to the rest of the training session. Decreases in RR interval, i.e., increase in HR, can arise from an increase in physical exertion and/or psychological stress, with negative correlations between intensity of exercise and overall HRV reported [5]. In the case of this study, the RR intervals recorded during training were representative of values that would be expected for low-medium exercise [5,26] and were within the range of values reported previously for initial training of horses [2,4,8,23,24] and more favourable to those reported for novel object/startle tests [16,17]. The significantly lower maximum RR observed during Join-up^®^ compared to the rest of the training session, is likely to represent an increase in HR due to horses exerting more physical activity during this training step which included short episodes of canter.

Time-domain variables SDRR, RMSSD, SD1, and SD2 were significantly higher in the stable compared to overall training, with no significant differences observed between Join-up^®^ and specific training. SDRR is recognized as a measurement of total power and as such it contains contributions from both the PNS and SNS and is strongly influenced by mean HR [6,27,28]. RMSSD reflects the high frequency beat-to-beat variations reflective of PNS activity [6,27,28]. SD1 and SD2 are mathematically related to SDRR and RMSSD [28]. SD1 is a primary variable used to estimate high frequency beat-to-beat variations and is known as short term HRV, SD2 represents long term changes in HR based on summation of successive RR intervals [6]. Decreases in SD1 tend to represent activation of SNS. SD2 represents long term changes in HR with increases indicative of SNS activity [6]. Based on these principles and the variables SDRR, RMSSD, and SD1, the data suggests that PNS dominance was reduced during overall training compared to activity within the stable. However, there is no evidence for any further reduction in PNS when Join-up^®^ is compared to specific training. However, because of the close interplay between the PNS and SNS, analysis of HR and time-domain measures of HRV to describe sympathetic-parasympathetic balance are not sufficient alone. An interesting observation is that during overall training (compared to stable) and Join-up^®^ (compared to specific training) SD2 is lowered. This implies that SNS activity is reduced (or inhibited) during Join-up^®^ (compared to specific training) and overall training (compared to stable). Within the literature this observation has been reported previously during the second training session of a week in initial training of horses [8], however, within this study the authors only comment on the increase in SD2 during the first training session as an indicator of increases in the SNS, but do not discuss why there was a decrease in SD2 observed in the second training session. This observation warrants further investigation in an appropriately controlled study design.

Frequency-domain analysis was performed to better understand the sympathetic-parasympathetic balance. The LF/HF ratio was significantly higher in the stable compared to overall training, with no significant differences observed in the LF/HF ratio between Join-up^®^ and specific training. HF power is an index of parasympathetic cardiac output, whilst the LF power is likely derived from both PNS and SNS [28]. The LF/HF ratio can, therefore, be used to indicate both sympathetic tone and cardiac sympathetic-parasympathetic balance [28]. Based on these principles, the data suggests that PNS dominance was reduced during overall training compared to activity within the stable. However, there is no evidence for any further reduction in PNS when Join-up^®^ is compared to specific training.

## 5. Conclusions

In conclusion, training of horses during public demonstrations is a low-moderate physiological stressor associated with reductions in PNS activity as indicated by changes in HR and HRV, however, the stress responses observed were comparable, or less than those reported in the literature for horses being used outside public events. Additionally, there is no evidence that the use of Join-up^®^, or other methods used by Monty Roberts, alter HR and HRV in a way to suggest that these training methods negatively affect the psychological welfare of horses. This study illustrates how the simple collection of HRV data during real-life training events can usefully inform our understanding of the welfare of horses. Further opportunistic studies encompassing different horsemanship styles and competitive disciplines would enable the generation of data which could complement that obtained during controlled scientific study.

## 6. Limitations

HR and HRV are influenced by a range of external variables including sex, age, breed, circadian rhythm, time since last meal, physical exertion, pain, and stress [6,27,28,29,30,31,32]. Because this study was opportunistic, collection of data from horses which were being used in prearranged public demonstrations, there are a number of limitations.

### 6.1. Sex, Age and Breed

Studies have shown that sex and age can affect HRV, with mares and young horses having greater HRV compared to geldings [30,31]. Because this study represented opportunistic data collection, it was not possible to study the HR and HRV response in a homogeneous group. Nevertheless, ‘sex’ was introduced as a fixed effect during statistical analysis with no differences observed in the HR and HRV response between mares and geldings. This however could be due to limited sample size, in particular that of the mares. In this study it was not possible to characterize horses according to age groups and therefore the effect of age was not determined. Breed has also been suggested to have a weak relationship to HRV [31], however, it was not possible to examine for a breed effect in this study because all horses were of a different breed type.

### 6.2. Circadian Rhythm

The baseline stable and training HR and HRV data were all collected between 7 p.m. and 11:30 p.m. Studies have shown that activity of the PNS is greatest during the evening [29], however, in this study it was not possible to compare HR and HRV data collected during night demonstrations compared to day demonstrations since all of Monty Roberts’ demonstrations were conducted at night.

### 6.3. Time Since Last Meal

Within this study, horses had access to *ad libitum* hay during transportation to the demonstration and from arrival until the point of leaving the stable for the arena. However, the actual consumption of hay within this study was not monitored. Studies have shown that fasting can lower HR and decrease LF/HF ratio meaning that parasympathetic activity is increased. It is possible that the eating patterns of horses used within this study were altered and may therefore have affected HR and HRV.

### 6.4. Physical Exertion

Changes in cardiac activity are strongly influenced by physical activity and/or psychological stress. As a result, analysis of HRV to determine autonomous system activity during intensive physical exertion is not suitable [5]. However, if monitors are used correctly to avoid introductions of artefacts associated with movement of electrodes, HRV analysis can be used to determine autonomous system activity during low exercise intensities [5,31]. To control for any artefacts in the data within this study, the custom filter was set to maximum for artefact correction in RR (ms). Horses’ baseline measurements were recorded within a stable environment where the physical activity was limited to what can be expressed within a standard loose box. While in the stable horses were only observed either standing eating hay or momentarily walking around the loose box. During training, horses were either stationary, walking, or trotting. Canter was only initiated purposefully during Join-up^®^ for short periods of time. Therefore, the physical exertion of horses being used within the demonstrations would be considered of low-medium exercise intensity. Research has shown that increasing fitness can influence LF/HF ratios [29]. In the case of this study there was no statistical difference between “starter horse” (no history of fitness training) and “remedial horse” (horses with a history fitness training).

### 6.5. Pain

Pain has the potential to influence HR and HRV [6,27], however, within this current study all horses were confirmed fit and well by appointed independent Veterinarians and Veterinary Physiotherapists prior to monitoring.

### 6.6. Other Variables Which May Affect HR and HRV Data Interpretation

Baseline stable recordings varied in length and were collected at the demonstration location rather than in familiar settings. In addition, it was not possible to standardize training methods used between horses, although the training steps used within the “starter” and “remedial” categories were similar, they were tailored to individual horses. Additionally, the location of the demonstrations varied, as did the treatment; some horses arrived on the day whilst some were already present. Future studies could aim to standardize these variables, use a within-horse study design and incorporate appropriate non-task controls [32] as the baseline. Furthermore, this study could be built upon by utilizing a range of additional measures of welfare, such as behavioural and salivary cortisol analysis to compliment HR and HRV and recording of exercise intensity/respiration rate to account for coupling between breathing frequency and stride and thus better inform interpretation of frequency-domain parameters.

Despite these limitations, this study is the first to report the HR and HRV of horses being used in Monty Roberts’ public demonstrations and provides an initial platform for discussion on this topic to build from.

## Figures and Tables

**Table 1:** Details of demonstration horses and demonstration location. Owners were asked whether their horses had been in a round pen or an arena before and whether they had experienced an audience. Horses were classified as (i) starter if they were going to experience their first saddle and rider during the demonstration; (ii) remedial if they were going to experience training associated with a problem behaviour.

Horse ID	Age (Years)	Demonstration Use	Breed	Sex	Transport Time to Demonstration	Time in Stable Prior to Monitoring	Demonstration Location	In Round Pen before	In Arena before	Audience before
A	3	Starter	Arab	Gelding	60 min	60–180 min	Hadlow	NO	NO	YES
B	3	Starter	TB × ID	Gelding	40 min	60–180 min	Reaseheath	NO	NO	NO
C	3	Starter	Sports Horse	Mare	30 min	60–180 min	Hartpury	NO	NO	NO
D	7	Remedial-Spooky	Warmblood	Mare	60 min	60–180 min	Hadlow	NO	YES	YES
E	11	Remedial-Spooky	Sports Horse	Gelding	Already on yard one plus week		Reaseheath	NO	YES	NO
F	2	Remedial-Loading	Cob × Trotter	Gelding	60 min	240–360 min	Hartpury	NO	NO	NO
G	12	Remedial-Loading	Cob	Mare	Already on yard one plus week		Hadlow	NO	YES	NO
H	4	Remedial-Head shy	Appaloosa	Gelding	60 min	60–180 min	Hartpury	NO	NO	NO
I	7	Remedial-Bike phobic	Haflinger	Gelding	45 min	60–180 min	Hartpury	YES	YES	YES
J	5	Remedial-Mounting	TB × ID	Gelding	90 min	60–180 min	Reaseheath	YES	YES	YES

TB: Thoroughbred; ID: Irish Draft; ×: cross-bred.

**Table 2:** Details of each training session.

Horse ID	Training Classification	Recording Time in Stable *	Training Steps	Training Time *
A	Starter	23:13	1. 1st Join-Up^®^, 2. First saddle plus 2nd Join-Up^®^, 3. First rider (Ardall dummy rider) plus 3rd Join-Up^®^ plus longlines, 4. First real rider (demo rider)	33:44
B	Starter	50:44	1. Headcollar (Dually™) schooling, 2. 1st Join-Up^®^, 3. First saddle plus 2nd Join-Up^®^, 4. First rider (Ardall dummy rider) plus 3rd Join-Up^®^, 5. First real rider (demo rider)	34:58
C	Starter	23:34	1. 1st Join-Up^®^, 2. First saddle plus 2nd Join-Up^®^, 3. First rider (Ardall dummy rider) plus 3rd Join-Up^®^ plus longlines, 4. First real rider (demo rider)	46:59
D	Remedial-Spooky	07:14	1. Plastic bag desensitization (plastic bag on end of stick), 2. Tarpaulin desensitization (free movement and leading over tarpaulin)	31:21
E	Remedial-Spooky	06:29	1. 1st Plastic bag desensitization (plastic bag on end of stick), 2. 1st Join-Up^®^, 3. 2nd Plastic bag desensitization (plastic bag on end of stick), 4. Tarpaulin desensitization (free movement and leading over tarpaulin)	29:55
F	Remedial-Loading	25:14	1. Head collar (Dually™) schooling, leading through open panels and over wooden board (ground schooling), 2. Trainer loading, 3. Owner loading	17:28
G	Remedial-Loading	06:17	1. Head collar (Dually™) schooling, 2. Leading through open panels and over wooden board (ground schooling), 3. Panels closed behind horse; trainer loads horse, 4. Trainer loading horse with open panels, 5. Owner loading horse with no panels	21:40
H	Remedial-Head shy	30:25	1. 1st Join-Up^®^, 2. Centaur Training	23:04
I	Remedial-Bike shy	18:23	1. 1st Join-Up^®^, 2. Horse tacked up, plastic bag desensitization (plastic bag on end of stick), 3. Ardall dummy legs, 4. Two bikes brought into round pen, horse moved freely forwards to follow bikes (bike desensitization)	32:06
J	Remedial-Mounting	12:01	1. Head collar (Dually™) schooling, 2. 1st Join-Up^®^, 3. Tacked up and horse moved forwards and backwards from the ground via head collar, 4. Rider (demo rider) on and horse moved forwards and backwards by mounting block; rider off and horse moved backwards, 5. Horse moved to side of mounting block; Rider (Owner) on	31:40

* Length of recording (in minutes) from mobile recording system (Polar RS800CX; Polar Electro Oy, Kempele, Finland).

## References

[1] Goodwin D., McGreevy P., Waran N., McLean A. (2009). How equitation science can elucidate and refine horsemanship techniques. Vet. J..

[2] Fowler V.L., Kennedy M., Marlin D. (2012). A comparison of the Monty Roberts training technique with a conventional UK technique for the initial training of riding horses. Anthrozoos.

[3] Kedzierski W., Janczarek I., Stachurska A. (2012). Emotional response of naïve Purebred Arabian colts and fillies to sympathetic and traditional training methods. J. Equine Vet. Sci..

[4] Visser E.K., Van Dierendonck M., Ellis A.D., Rijksen C., Van Reenen C.G. (2009). A comparison of sympathetic and conventional training methods on response to initial horse training. Vet. J..

[5] Physick-Sheard P.W., Marlin D.J., Thornhill R., Schroter R.C. (2000). Frequency domain analysis of heart rate variability in horses at rest and during exercise. Equine Vet. J..

[6] Von Borell E., Langbein J., Després G., Hansen S., Leterrier C., Marchant-Forde J., Marchant-Forde R., Minero M., Mohr E., Prunier A. (2007). Heart rate variability as a measure of autonomic regulation of cardiac activity for assessing stress and welfare in farm animals—A review. Physiol. Behav..

[7] Task Force of the European Society of Cardiology, The North American Society of Pacing and Electrophysiology (1997). Heart rate variability: Standards of measurement, physiological interpretation and clinical use. Comment in Circulation. Eur. Heart J..

[8] Schmidt A., Aurich J., Möstl E., Müller J., Aurich C. (2010). Changes in cortisol release and heart rate and heart rate variability during the initial training of 3-year-old sport horses. Horm. Behav..

[9] Janczarek I., Stachurska A., Kędzierski W., Wilk I. (2013). Responses of horses of various breeds to a sympathetic training method. J. Equine Vet. Sci..

[10] Munsters C.C.B.M., van den Broek J., van Weeren R., Sloet van Oldruitenborgh-Oosterbaan M.M. (2013). The effects of transport, riot control training and night patrols on the workload and stress of mounted police horses. Appl. Anim. Behav. Sci..

[11] Munsters C., Visser K., van den Broek J., Sloet van Oldruitenborgh-Oosterbaan M.M. (2013). Quantifying stress in experienced and inexperienced mounted police horses, using heart rate, heart rate variability, behavior score and suitability score. J. Vet. Behav. Clin. Appl. Res..

[12] Von Lewinski M., Biau S., Erber R., Ille N., Aurich J., Faure J.M., Möstl E., Aurich C. (2013). Cortisol release, heart rate and heart rate variability in the horse and its rider: Different responses to training and performance. Vet. J..

[13] Kinnunen S., Laukkanen R., Haldi J., Hanninen O., Atalay M. (2006). Heart rate variability in trotters during different training periods. Equine Vet. J. Suppl..

[14] Munsters C.C.B.M., Visser K.E.K., van den Broek J., Sloet van Oldruitenborgh-Oosterbaan M.M. (2012). The influence of challenging objects and horse-rider matching on heart rate, heart rate variability and behavioural score in riding horses. Vet. J..

[15] Rietmann T.T., Stuart A.E.A., Bernasconi P., Stauffacher M., Auer J.A., Weishaupt M.A. (2004). Assessment of mental stress in warmblood horses: Heart rate variability in comparison to heart rate and selected behavioural parameters. Appl. Anim. Behav. Sci..

[16] Villas-Boasa J.D., Diasb D.P.M., Trigoc P.I., dos Santos Almeidaa N.A., de Almeidac F.Q., de Medeirosa M.A. (2015). Behavioural, endocrine and cardiac autonomic responses to a model of startle in horses. Appl. Anim. Behav. Sci..

[17] Visser E.K., van Reenen C.G., van der Werf J.T., Schilder M.B., Knaap J.H., Barneveld A., Blokhuis H.J. (2002). Heart rate and heart rate variability during a novel object test and a handling test in young horses. Physiol. Behav..

[18] Schmidt A., Hödl S., Möstl E., Aurich J., Müller J., Aurich C. (2010). Cortisol release, heart rate, and heart rate variability in transport-naive horses during repeated road transport. Domest. Anim. Endocrinol..

[19] Schmidt A., Möstl E., Wehnert C., Aurich J., Müller J., Aurich C. (2010). Cortisol release and heart rate variability in horses during road transport. Horm. Behav..

[20] Erber R., Wulf M., Aurich J., Rose-Meierhöfer S., Hoffmann G., von Lewinski M., Möstl E., Aurich C. (2013). Stress response of three-year-old horse mares to changes in husbandry system during initial equestrian training. J. Equine Vet. Sci..

[21] Roberts M. (2002). 1935- From My Hands to Yours: Lessons from a Lifetime of Training Championship Horses.

[22] McGreevy P.D., McLean A.N. (2007). Roles of learning theory and ethology in equitation. J. Vet. Behav..

[23] Wilk I., Janczareka I. (2015). Relationship between behaviour and cardiac response to round pen training. J. Vet. Behav..

[24] Wilk I., Kędzierskib W., Stachurskaa A., Janczareka I. (2015). Are results of Crib Opening Test connected with efficacy of training horses in a round-pen?. Appl. Anim. Behav. Sci..

[25] Becker-Birck M., Schmidt A., Lasarzik J., Aurich J., Möstl E., Aurich C. (2013). Cortisol release and heart rate variability in sport horses participating in equestrian competitions. J. Vet. Behav..

[26] Aerts J.M., Gebruers F., Van Camo E., Berckmans D. (2008). Controlling horse heart rate as a basis for training improvement. Comput. Electron. Agric..

[27] Stucke D., Große Ruse M., Lebelt D. (2015). Measuring heart rate variability in horses to investigate the autonomic nervous system activity—Pros and cons of different methods. Appl. Anim. Behav. Sci..

[28] Brennan M., Palaniswami M., Kamen P. (2001). Do existing measures of Poincaré plot geometry reflect nonlinear features of heart rate variability?. IEEE Trans. Biomed. Eng..

[29] Kuwahara M., Hiraga A., Kai M., Tsubone H., Sugano S. (1999). Influence of training on autonomic nervous function in horses: Evaluation by power spectral analysis of heart rate variability. Equine Vet. J. Suppl..

[30] Bowen I.M., Marr C.M. (2010). Ambulatory electrocardiography and heart rate variability. Cardiology of the Horse.

[31] Clément F., Barrey E. (1995). Heart rate fluctuations in the horse at rest: (2) Biological variation factors related to behavioural profile. Comptes Rendus Acad. Sci. Ser. III Sci. Vie.

[32] Quintana D.S., Heathers J.A. (2014). Considerations in the assessment of heart rate variability in biobehavioral research. Front. Psychol..

